# Apoptosis-Related Factors in the Luteal Phase of the Domestic Cat and Their Involvement in the Persistence of *Corpora Lutea* in Lynx

**DOI:** 10.1371/journal.pone.0143414

**Published:** 2015-11-24

**Authors:** Olga Amelkina, Lina Zschockelt, Johanna Painer, Rodrigo Serra, Francisco Villaespesa, Beate C. Braun, Katarina Jewgenow

**Affiliations:** 1 Department of Reproduction Biology, Leibniz Institute for Zoo and Wildlife Research, Berlin, Germany; 2 Iberian lynx captive breeding program, Centro Nacional de Reprodução de Lince Ibérico, Silves, Portugal; 3 Iberian lynx captive breeding program, Centro de Cría de Lince Ibérico El Acebuche, Parque Nacional de Doñana, Huelva, Spain; Justus-Liebig-Universität, GERMANY

## Abstract

The *corpus luteum* (CL) is a transient gland formed in the ovary after ovulation and is the major source of progesterone. In the Iberian and Eurasian lynx, CL physiologically persist after parturition and retain their capacity to produce progesterone, thus suppressing the ovarian activity. This unique reproductive characteristic has a big impact on the success of assisted reproduction techniques in the endangered Iberian lynx. The mechanisms behind CL persistence are not yet understood and require extensive studies on potential luteotropic and luteolytic factors in felids. Because the apoptosis system has been shown to be involved in structural regression of CL in many species, we aimed to investigate the capacity of perCL to undergo apoptosis. In addition, we performed initial studies on the apoptosis system in the luteal phase of the domestic cat. No previous research on this system has been made in this species. Our factors of interest included agents of the intrinsic apoptosis pathway, *i*.*e*., pro-survival B-cell CLL/lymphoma 2 (BCL2) and pro-apoptotic BCL2-associated X protein (BAX), the executioner caspase-3 (CASP3), as well as of the extrinsic pathway, *i*.*e*., pro-apoptotic receptor FAS, and tumor necrosis factor (TNF) and its receptors (pro-apoptotic TNFRSF1A and pro-survival TNFRSF1B). We analyzed the relative mRNA levels of these factors, as well as protein localization of CASP3 and TNF during stages of pregnancy and the non-pregnant luteal phase in CL of domestic cats. The same factors were investigated in freshly ovulated CL (frCL) and perCL of Iberian and Eurasian lynx, which were histologically analyzed. All factors were present in the CL tissue of both domestic cat and lynx throughout all analyzed stages. The presence of pro-apoptotic factors *BAX*, *CASP3*, *FAS* and *TNFRSF1A* in perCL of the Eurasian and Iberian lynx might indicate the potential sensitivity of perCL to apoptotic signals. The expression of pro-survival factors *BCL2* and *TNFRSF1B* was significantly higher in perCL compared to frCL of studied Iberian lynx, suggesting the potential involvement of these factors in the structural integrity of perCL. In both Iberian lynx and pregnant and non-pregnant domestic cats, the expression of *TNFRSF1A* was significantly higher in forming CL compared to other stages, suggesting the conserved involvement of this factor in the tissue reorganization during formation of the feline CL. The mRNA levels of *CASP3* and *TNFRSF1B* were highest during regression stages of domestic cat CL. The current study provides initial results on the possible involvement of the apoptosis system in the structure and function of the feline CL and in its physiological persistence.

## Introduction

The *Lynx* genus includes four species that inhabit different parts of the world: the Eurasian lynx (*Lynx lynx*) in the broad range of Europe and Asia, the Canada lynx (*Lynx canadensis*) and the bobcat (*Lynx rufus*) in North America, and the Iberian lynx (*Lynx pardinus*) in the Iberian Peninsula. Eurasian, Canada and Iberian lynx are monoestrous, *i*.*e*., mate only once in a year, with breeding seasons in January–April, February–April and January–February, respectively [1–4]. The bobcat is seasonally polyestrous and may ovulate up to three times during the breeding season [5].

All three monoestrous lynx species exhibit a unique reproductive characteristic associated with the *corpus luteum* (CL), a transient gland that forms in the ovary after ovulation and supports pregnancy *via* its production of progesterone [6]. It has been widely shown among different species that, in the event of a non-pregnant cycle or at the end of pregnancy, the CL regresses from the ovarian tissue and a new cycle is initiated [7, 8]. However, the situation is markedly different for the lynx. Studies on the lynx ovarian cycle revealed the presence of CL outside the reproductive season in Iberian and Eurasian lynx [9]. More recent findings indicate that CL of the Eurasian lynx morphologically persist in the ovary for at least two years [10]. Furthermore, such persistent CL (perCL) continue to produce progesterone and thus exhibit functional persistence, leading to a stage called prolonged diestrus between cycles [10, 11]. Studies on the Canada lynx also suggest a similar luteal pattern, based on constant fecal progesterone concentrations [1]. The bobcat seems to exhibit morphological persistence of CL, however, their functional condition still needs to be investigated [12, 13]. To summarize, CL persist morphologically and functionally after parturition and weaning in all three monoestrous lynx species: Iberian, Eurasian and Canada lynx.

There is as yet no certainty about the role of perCL in lynx reproduction, nor are there any reports so far on the possible molecular mechanisms involved. It has been hypothesized that perCL may be advantageous in lynx species by supporting subsequent pregnancies as additional sources of progesterone [14] and/or by securing a monoestrous cycle *via* progesterone suppression of ovarian activity [15]. Such suppression of any late ovulations would ensure the birth and weaning of cubs during the most favorable time of the year [10]. It is unclear how the monoestrous lynx enters a new ovarian cycle every year despite the constant presence of active perCL and, therefore, it is not possible to design any protocols of ovulation induction for these species. An attempt to overcome the CL persistence by administrating a commonly luteolytic prostaglandin F_2α_ led to a temporary decrease in progesterone concentration, but structurally perCL remained in the ovary [16]. The lynx unique reproductive characteristic, therefore, makes standard protocols for assisted reproduction inapplicable in monoestrous lynx and may critically lower the success of breeding programs for these species. This greatly affects the Iberian lynx, which until recently had been listed as critically endangered by the International Union for Conservation of Nature [17] and for which extensive genetic studies revealed a high extinction risk of the two remaining populations [18, 19]. To support and restore wild populations of the Iberian lynx, a captive breeding program has been initiated in the centers of Spain and Portugal with ongoing breeding and reintroduction of animals [20]. Unraveling the function and regulation of perCL, and particularly the mechanisms of its regression, could enhance the performance of captive breeding programs and offer more opportunities for the Iberian lynx survival.

The research on the lynx or any other wild felid is complicated by the limited access to these species. The sampling of reproductive tissue is rare and depends heavily on a chance, be it *post-mortem* sampling after legal hunting or natural/accidental death of an animal, or an ovariohysterectomy of captive lynx for medical and management reasons. Because of this, the more accessible felid, domestic cat (*Felis catus*), becomes an irreplaceable model species. The domestic cat is seasonally polyestrous, and after ovulation queens can either enter a period of pregnancy (approximately 65 days [21]) or a non-pregnant luteal phase (approximately 40 days [22]). In both scenarios, elevated serum and intraluteal progesterone concentrations decrease towards the end of the luteal phase, allowing initiation of a subsequent ovarian cycle [23, 24]. Molecular studies on the domestic cat CL have been limited to the investigation of enzymes of the luteal steroidogenesis system [25]. To contribute to the knowledge on the mechanisms behind formation, maintenance and regression of the feline CL, we initiated a detailed molecular study on potential luteotropic and luteolytic factors in the domestic cat [26] and lynx [27]. In the present work, we focus on the mechanisms of structural regression of the CL in the domestic cat and in two species of lynx, Iberian and Eurasian.

Luteal regression (luteolysis) is commonly required for the initiation of a new follicular cycle. The literature distinguishes between two aspects of luteolysis, functional and structural, with the first condition usually preceding the latter one [8]. Both aspects of luteolysis are governed by species-specific luteal factors, however, structural luteal regression *via* apoptosis-related factors is common to most of the species studied to date [7, 28–30]. Apoptosis, or programmed cell death, can be divided into intrinsic and extrinsic pathways. The intrinsic signaling cascade is generally activated by apoptotic stimuli within a cell. Members of the B-cell CLL/lymphoma 2 (Bcl-2) family transduce the signal within the cell by alterations in the mitochondrial outer membrane (MOM): *e*.*g*., the pro-apoptotic factor BCL2-associated X protein (BAX) resides in the cytosol or on MOM itself and, once activated by the BH3-only proteins and oligomerized, creates permeability of the MOM [31, 32]. This permeability leads to release of cytochrome c from the inner mitochondrial membrane and thus causes activation of caspase-3 (CASP3) which, through cleavage of essential proteins, executes the final step of cell death [33]. The pro-survival factor BCL2 can inhibit the death signal *via* translocation of BAX from MOM, or by sequestering BH3-only proteins and preventing them from activating BAX, or by binding to activated BAX and preventing its oligomerization [31]. All the mentioned factors were shown to be involved in luteolysis in rodents, cattle and primates [34–39].

The extrinsic signaling cascade is activated by extracellular signals *via* cytokines, which include members of the tumor necrosis factor (TNF) super family, *e*.*g*., TNF (also known as TNF superfamily, member 2 and TNF-alpha) and Fas ligand, and their cell surface receptors, *e*.*g*., TNF receptor superfamily, member 1A (TNFRSF1A; previously known as TNFR1), TNFRSF1B (previously known as TNFR2) and Fas cell surface death receptor (FAS; also known as CD95) [40]. The FAS/Fas ligand system is known to transduce the death signal *via* protease cascade and was shown to be required for luteolysis in many species [41–44]. Another cytokine, TNF, exhibits pleiotropic signals in the CL tissue and its action might be dependent on its concentration, the local environment, the stage of the cycle and the type of receptor it binds to [43, 45–47]. While TNFRSF1A contains the death domain and can transduce the death signal, in contrast, TNFRSF1B does not possess the death domain and can act as a pro-survival factor [40, 48, 49]. Presented list of factors was found to play an important role throughout the luteal phase of many species, including representatives of rodents, cattle and primates [42, 48–53]. Both intrinsic and extrinsic pathways lead to activation of the caspase family, *i*.*e*., caspase-9 and -8 respectively, and subsequently the final executioners caspase-3, -6 and -7 [54]. Short description of each factor can be found in Table 1.

**Table 1:** List of analyzed factors, sequences of PCR primers used for sequence analysis and expression studies, annealing temperatures, and product sizes. bp, base pair; fw, forward; rv, reverse; T_A_, annealing temperature; a, used for sequence analysis; b, used for gene expression studies. Intrinsic/extrinsic pathway refers to the apoptosis pathways

Factor	Short description	GenBank ID	Species	Primer sequence 5’– 3’	T_A_ (°C)	Product size (bp)	Use
*BCL2*	Bcl-2 family, intrinsic pathway	DQ926871.1 KP826765*	*Felis catusLynx pardinus*	*BCL2* Fw: GAG ATG TCC AGC CAG CTG*BCL2* Rv: TAG GCA CCC AGG GTG ATG	53	365	a
	pro-survival function			*BCL2* qFw: GGA GGA TTG TGG CCT TCT*BCL2* qRv: GGT TCA GGT ACT CAG TCA TCC AC	54.5	143	b
*BAX*	Bcl-2 family, intrinsic pathway	DQ926869.1KP862666*	*Felis catusLynx pardinus*	*BAX* Fw: CAG CTC TGA GCA GAT CAT G*BAX* Rv: TGG TGG CCT CAG CCC ATC T	53	595	a
	pro-apoptotic function			*BAX* qFw: CCG ATG GCA ACT TCA ACT GGG*BAX* qRv: GAT GGT CAC TGT CTG CCA CGT C	63	244	b
*CASP3*	protease, intrinsic pathway	NM_001009338.1KP294340*	*Felis catusLynx pardinus*	*CASP3* Fw: GTG TGC GTT AGA AGT ACC*CASP3* Rv: GTT CTT TTG TGA GCA TAG ACA	53	836	a
	executioner function			*CASP3* qFw: ACC GGC AAA CCC AAA CTC*CASP3* qRv: CTG ACA GGC GAT GTC ATC C	60.5	91	b
*FAS*	cytokine receptor, extrinsic pathway	NM_001009314.1KP670850*	*Felis catusLynx pardinus*	*FAS* Fw: CCG TTG GCT GAT ACT TAC C*FAS* Rv: CGT GTT TGC AGT TTC CAT TC	53	952	a
	generally pro-apoptotic function			*FAS* qFw: GAA CGC TAC CGA AGG GGA*FAS* qRv: GTC GGC AGC TTT TCG TGT	59.5	100	b
*TNFRSF1A*	cytokine receptor, extrinsic pathway	NM_001009361.1KP670852*	*Felis catusLynx pardinus*	*TNFRSF1A* Fw: GCA GGA AGA ACC AGT ACC GG*TNFRSF1A* Rv: CCG TTC TGC AGC TCC AGC C	53	846	a
	pro-apoptotic function			*TNFRSF1A* qFw: AGA GTG TAC GAA GTT GTG CG*TNFRSF1A* qRv: AGC TTG GAC TTC CGC CGT T	60.5	159	b
*TNFRSF1B*	cytokine receptor, extrinsic pathway	KP670853*KP670854*	*Felis catusLynx pardinus*	*TNFRSF1B* Fw: CGC CCG GGC TTC GGC G*TTNFRSF1B* Rv: CTT GGA GAA GGG GAC CTG CT	53	799	a
	pro-survival function			*TNFRSF1B* qFw: AGC AGC TCC CTG GAG AGC *TTNFRSF1B* qRv: GTG ACA TTG ACC TGG GTC C	60.5	168	b
*TNF*	cytokine, extrinsic pathway	NM_001009835.1KP670851*	*Felis catusLynx pardinus*	*TNF* Fw: CAT GAG CAC TGA AAG CAT GA*TNF* Rv: TCA CAG GGC AAT GAT CCC A	53	703	a
	pleiotropic effects			*TNF* qFw: AGA GCT CCC ACA TGG CCT*TNF* qRv: GGC TCA GCC ACT GGA GTT	59.5	137	b

* GenBank sequences were obtained in this study

We had three hypotheses that might explain the prolonged structural integrity of perCL in lynx: (i) either CL persist due to their limited capacity to undergo apoptosis; or (ii) there is no luteolytic signal and, therefore, no activation of the apoptotic cascade; or (iii) the transducing luteolytic signal is blocked on the downstream of the apoptotic cascade. To study these potential mechanisms, we aimed to first investigate the capacity of perCL to undergo apoptosis. As a necessary basis, we studied the involvement of selected apoptosis-related factors, *i*.*e*., *BCL2*, *BAX*, *CASP3*, *FAS*, *TNFRSF1A*, *TNFRSF1B* and *TNF*, in the luteal phase of the domestic cat. Then, we analyzed the presence of these factors in perCL of Iberian and Eurasian lynx and compared the capacity of freshly ovulated (fr) CL and perCL to express them in the Iberian lynx.

## Materials and Methods

All chemicals in the study were purchased from Sigma-Aldrich (Taufkirchen, Germany), unless stated otherwise and were of the highest purity available.

### 2.1 Ethics statement

The methods applied, and the study-design, were approved by the Internal Committee for Ethics and Animal Welfare of the Leibniz Institute for Zoo and Wildlife Research in Berlin, Germany (Permit numbers: 2010-10-01 and 2011-01-01). The Norwegian Experimental Animal Ethics Committee approved the collection of the ovarian tissue from hunted animals (Permit number: 2010/161554). The tissue from late pregnancy castration of domestic cats were obtained from the Institute of Animal Reproduction and Food Research of the Polish Academy of Sciences, Olsztyn, Poland, and all procedures were approved by the Local Animal Care and Use Committee in Olsztyn, Poland (No. 41/2007/N and 61/2010/DTN).

### 2.2 Animals and tissue collection

Ovaries of domestic cats were obtained from local animal shelters and clinics after ovariohysterectomy for the purpose of permanent contraception. The reasons to perform ovariohysterectomy were not related to the study. Samples were transported in MEM-HEPES medium, supplemented with 3 mg/mL BSA (Merck Millipore, Darmstadt, Germany) and 1x Antibiotic Antimycotic Solution. Transportation was at 4°C, and ovaries were processed immediately after arrival at the laboratory (2–4 hours after surgery). The isolation process and consequent staging of CL is described in Amelkina *et al*. [24]. In brief, CL from each cat were either fixed in Bouin’s solution for histologic analysis or plunged into liquid nitrogen for RNA isolation. In the case of pregnancy, the day was assessed by the diameter of the gestation chamber [55], the crown-rump length of a foetus [56] or by the stage of a pre-implantation embryo [57]. The pre-implantation period (n = 6) included samples from days 2 to 6 and day 10 *post-coitum*; the post-implantation period (n = 11) included samples from days 14 to 36 *post-coitum*; finally, the CL regression stage (n = 5) was represented by samples from days 38, 39, 48 and week 9 *post-coitum*. The absence of embryos in the oviducts or uteri indicated a non-pregnant luteal phase. In such cases, based on their histologic appearance, each CL was classified as the stage of: formation (n = 9), development/maintenance (n = 13), early regression (n = 14), late regression (10) or *corpus albicans* (CA; n = 4). The histologic classification is described in detail in Amelkina *et al* [24] and includes parameters of: cell shape, type and degree of vacuolation, nucleus condition, and the ratio of non-steroidogenic to luteal cells. Listed n-values represent the number of animals per analyzed stage; each animal is represented by one CL.

Ovaries of Iberian lynx were collected in the scope of the Iberian lynx captive breeding program at the Centro de Cría de Lince Ibérico El Acebuche, Parque Nacional de Doñana, Huelva, Spain and Centro Nacional de Reprodução do Lince Ibérico, Silves, Portugal. Two animals were ovariohysterectomized for permanent contraception seven days after ovulation was induced by natural mating in February, 2013. Ovariohysterectomy was initiated due to the medical conditions of animals (repeated caesarean sections and mammary tumor risk) and was not related to the study. In one animal (Iberian lynx 1, nine years old), embryos were flushed from the uterus, indicating the pre-implantation stage of pregnancy. Unfertilized oocytes were flushed from the second animal (Iberian lynx 2, eleven years old), thus indicating a non-pregnant luteal phase. The CL were isolated immediately after surgery and their morphological appearance was noted. The presence of ovulation scars allowed distinguishing CL of fresh ovulation (frCL) from CL of previous cycle/s (perCL), supplemented later by histologic analysis of both types (see Results). Each CL was dissected and pieces were fixed in Bouin’s solution (for histologic analysis) or placed in RNA-later solution (RNA isolation; Qiagen GmbH, Hilden, Germany) or liquid nitrogen (hormone analysis).

Ovaries of free-ranging Eurasian lynx were collected freshly *post-mortem* from animals hunted legally during the national hunting quota for management purposes in Norway (n = 5 animals). The period of collection was the beginning of the breeding season prior to mating (February, 2011; the breeding season for Eurasian lynx in Norway is February to early April [2]). After dissection, samples were immediately fixed in Bouin’s solution (for histologic analysis) or placed in RNA-later (RNA isolation) or Allprotect Tissue Reagent (hormone analysis; Qiagen GmbH, Hilden, Germany) solutions. Based on the pre-breeding period of collection and the absence of frCL, embryos and placental scars of a recent pregnancy, all isolated CL were classified as perCL from previous cycle/s.

To demonstrate the steroidogenic activity of the isolated CL, intraluteal concentrations of progesterone and estrogens were determined for Iberian and Eurasian lynx by enzyme immunoassay per wet weight of CL, as described and validated previously [11, 24].

### 2.3 RNA isolation and cDNA synthesis

Up to 26 mg of CL tissue was homogenized in homogenization tubes (100 μl RNA lysis buffer, 1.4/2.8 mm ceramic beads) at 5000 rpm for 2 x 25 sec (Precellys 24 homogenizer, Bertin Technologies, Montigny-le-Bretonneux, France). Total RNA was extracted using the innuSPEED Tissue RNA/innuPREP DNase I Digest Kit (Analytik Jena AG, Jena, Germany). The NanoDrop ND-1000 (PEQLAB Biotechnologie GmbH, Erlangen, Germany) was used to assess the concentration and purity of isolated RNA. Additional control of RNA quality and integrity was performed *via* microfluidic analysis using the Bioanalyzer (Agilent Technologies Deutschland GmbH, Boeblingen, Germany); RNA integrity number (RIN) values were above 7.0 for cat samples and above 6.1 for Iberian and Eurasian lynx samples. From 1 to 2.5 μg of isolated RNA was reverse transcribed into single-stranded (ss) cDNA using the RevertAid First Strand cDNA Synthesis Kit (Thermo Fisher Scientific, Waltham MA, USA). No reverse transcriptase was added to the negative control to verify the absence of genomic DNA contamination.

### 2.4 Sequencing

Primers for the polymerase chain reaction (PCR) were purchased from BioTeZ Berlin Buch GmbH (Berlin, Germany) and were designed based on *Felis catus* gene sequences listed in NCBI database (see accession numbers in Table 1). The NCBI-sequence for *TNFRSF1B* was only predicted by automated computational analysis (XM_003989583.2) and thus it was analyzed in this study too. Primer information is listed in Table 1. Based on feline ss cDNA templates of luteal, ovarian or placental origin, partial cat and lynx cDNA sequences were amplified using the Expand High FidelityPLUS PCR system (Roche Diagnostics Deutschland GmbH, Mannheim, Germany), as described by Braun *et al*. [58]. For both cat and lynx, the PCR conditions were: 94°C for 2 min; followed by 35 cycles of denaturation at 94°C for 45 sec, annealing at 53°C for 45 sec, elongation at 72°C for 80 sec; and final elongation at 72°C for 7 min. Purified PCR products were ligated to the pJET 1.2 vector (Thermo Fisher Scientific) and transfected in JM109 cells (Promega GmbH, Mannheim, Germany) for cat *CASP3*, *FAS* and *TNF* or ligated to the pCR4-TOPO TA vector and transfected in TOP10 cells (both Life Technologies GmbH, Darmstadt, Germany) for lynx *CASP3*, *FAS*, *TNF* and cat/lynx *TNFRSF1A* and *TNFRSF1B*. Positive clones or purified PCR products (*BCL2* and *BAX*) were sequenced by the Services in Molecular Biology GmbH (Dr M. Meixner, Brandenburg, Germany).

### 2.5 Quantitative real-time PCR

Primers for quantitative real-time PCR (qPCR) were based on cat and lynx sequences identified in this study (Table 1). The qPCR was performed using the CFX96 Real-Time PCR Detection System (Bio-Rad Laboratories GmbH, Munich, Germany), as published by Braun *et al*. [59]. In brief, diluted ss cDNA (4 μl, corresponding to 2 or 10 ng of total RNA for genes of interest, or 4 ng for reference genes) were analyzed in a 10 μl reaction volume including SsoFast EvaGreen Supermix (Bio-Rad Laboratories GmbH). The qPCR conditions were: 98°C for 2 min and 40 cycles of 8 sec at 98°C and 8 sec at different annealing temperatures (Table 1). Quantification of qPCR products was performed using the CFX Manager Software 1.6 (Bio-Rad Laboratories GmbH). Serial dilutions of plasmid DNA were used for calibration. Glutaminase (*GLS*; for domestic cat, JQ424891), TATA box binding protein (*TBP*; for domestic cat, JQ424890; for lynx, JX993351), β-actin (*BACT*; for domestic cat, AB051104; for lynx, KM458620), glyceraldehyde 3-phosphate dehydrogenase (*GAPDH;* for lynx, KM458621) and ribosomal protein S7 (*RPS7*; for lynx, JX993349) were validated as optimal reference genes in feline CL with the qbasePLUS software (Biogazelle, Zwijnaarde, Belgium; [60]) and were used for normalization. A multiple normalization factor was calculated for individual CL referring to Vandesompele *et al*. [61].

### 2.6 Histologic analysis of CL and protein localization

Tissues fixed in Bouin’s solution were dehydrated, embedded in paraffin following standard procedures and sectioned at 3 μm. Subsequent histologic evaluation was performed under a light microscope fitted with a digital camera (Jenoptik ProgRes C3, Jena, Germany).

To determine the histomorphological state of CL, samples from Iberian and Eurasian lynx were routinely stained with haematoxylin and eosin. The histomorphological analysis of cat samples was performed in our previous study [24].

The localization of CASP3 protein in luteal tissue was assessed by an affinity-purified rabbit anti-human/mouse CASP3 reactive antibody (Cat. AF 835; R&D Systems, Wiesbaden, Germany), used in a previous study on the domestic cat [62]. According to the manufacturer, this antibody recognizes an active form of CASP3. Localization of TNF protein was assessed by a goat polyclonal anti-human TNF antibody (Cat. No. sc-1350; Santa Cruz Biotechnology, Dallas TX, USA). Immunohistochemistry was performed as described in Braun *et al* [58]. In brief, sectioned CL tissue mounted on microscope slides (Superfrost Plus, Thermo Scientific, Braunschweig, Germany) was deparaffinized in Roti-Histol (Carl Roth GmbH, Karlsruhe, Germany) and rehydrated in decreasing concentrations of ethanol. Slides were subsequently incubated in boiling citrate buffer (11 mM, pH 6.0) for 15 min and in 3% H_2_O_2_/methanol solution for 10 min. Then, sections were blocked with 5% BSA in PBS for 1 h at 37°C. Antibody against CASP3 was diluted in PBS 1:500; antibody against TNF was diluted in 1% BSA in PBS 1:200. Sections were subsequently washed with PBS-Tween 0.1%, incubated with either peroxidase-conjugated anti-rabbit or anti-mouse EnVisionC reagent (ready to use solution; Dako Agilent Technologies, Glostrup, Denmark) for 1 h at 23°C and colour-detected with diaminobenzidine (DAB) substrate chromogen solution (Dako Agilent Technologies). Finally, sections were counterstained with hematoxylin, dehydrated in increasing concentrations of ethanol, and covered with mounting medium and coverslips.

### 2.7 Statistical analysis

Statistical analysis was performed with the R software package (R: A language and environment for statistical computing, version 3.0.0, Vienna, Austria). For the domestic cat, the Kruskal-Wallis rank sum test was used to determine changes in relative mRNA levels throughout pregnant and non-pregnant luteal phases. The Wilcoxon rank sum test was used for *post-hoc* pairwise comparison of stages (P-value adjustment: Benjamini-Hochberg). Each animal was represented by one CL. Numbers of animals per stage can be found above in 2.2. The stage of CA was not included in the statistical analysis, because its origin (pregnant or non-pregnant luteal phase) in the ovary was unknown. For Iberian lynx, the Mann-Whitney U-test was used to determine changes in relative mRNA levels between frCL and perCL in each animal. Sample size for Iberian lynx was the following: Iberian lynx 1, n = 3 for frCL and n = 8 for perCL; Iberian lynx 2, n = 5 for frCL and n = 6 for perCL. Probability (P) values less than 0.05 were considered statistically significant. SigmaPlot 10.0 (Systat Software Inc., San Jose CA, USA) was used to visualize the statistical results *via* boxplots.

## Results

In this study, we obtained partial feline gene sequences for the analyzed factors (Table 1).

### 3.1. Domestic cat

#### 3.1.1 Pregnancy

**Fig 1:**
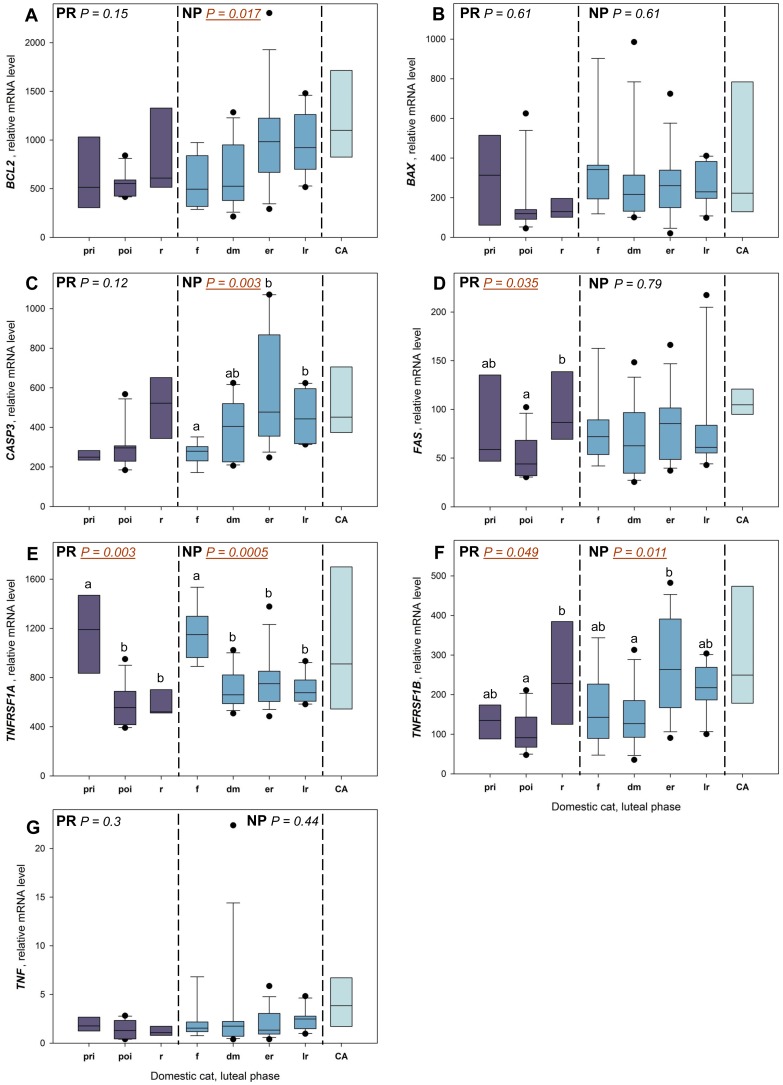
Intraluteal relative mRNA levels during pregnant and non-pregnant luteal phases in the domestic cat. Box plots depict the distribution of data; whiskers indicate maximum and minimum values; black dots indicate outliers; horizontal lines indicate the median; P-values are calculated from the Kruskal-Wallis rank sum test; letters (a, b) identify significant differences between stages and are calculated from *post-hoc* pairwise comparisons (P-value adjustment: Benjamini-Hochberg). PR: pregnancy; NP: non-pregnant luteal phase; pri: pre-implantation period; poi: post-implantation period; r: regression; f: formation; d/m: development/maintenance; er: early regression; lr: late regression; CA: *corpus albicans*.

Throughout the pregnant luteal phase, no significant changes were observed in mRNA levels of *BCL2*, *BAX*, *CASP3* and *TNF* (Fig 1A–1C/1G). Relative *FAS* mRNA levels were higher during CL regression compared to the post-implantation period (P = 0.026; Fig 1D). Gene expression of TNF receptors changed significantly during pregnancy: *TNFRSF1A* (Fig 1E) relative mRNA levels were higher during the pre-implantation period compared to post-implantation (P = 0.0019) and CL regression (P = 0.013); *TNFRSF1B* (Fig 1F) relative mRNA levels were higher during CL regression compared to post-implantation (P = 0.055).

#### 3.1.2 Non-pregnant luteal phase

Throughout the non-pregnant luteal phase, relative mRNA levels of *BCL2*, *CASP3*, *TNFRSF1A* and *TNFRSF1B* changed significantly, while no significant changes were observed for *BAX*, *FAS* and *TNF* (Fig 1). For *BCL2* (Fig 1A), relative levels of mRNA changed throughout the luteal phase (P = 0.017), but no significance was found in the *post-hoc* comparison. For *CASP3* (Fig 1C), relative mRNA levels during CL formation were lower compared to early regression (P = 0.0014) and late regression (P = 0.0038). Relative mRNA levels of *TNFRSF1A* (Fig 1E) were higher during CL formation compared to development/maintenance (P = 0.00023), early regression (P = 0.0013) and late regression (P = 0.00023). Finally, relative mRNA levels of *TNFRSF1B* (Fig 1F) during CL early regression were higher than during development/maintenance (P = 0.028).

**Fig 2:**
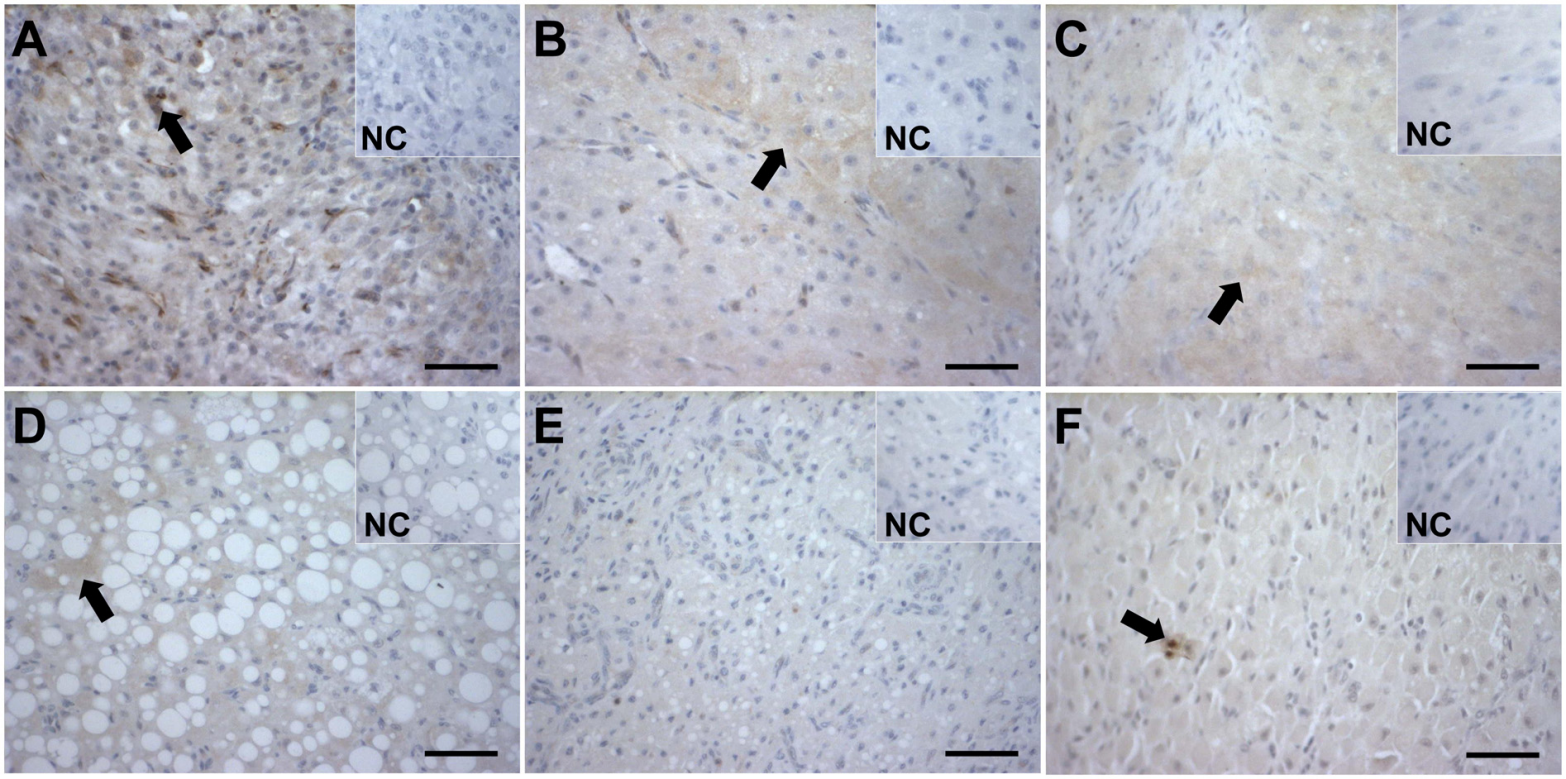
Immunohistochemical localization of CASP3 and TNF proteins in CL of the domestic cat. Active CASP3 was localized in luteal cells at the stages of: CL formation (A), development/maintenance (B), early regression (C) and late regression (D). The protein expression was consistently present throughout the CL life span, except for CA (E). TNF protein expression was identified in non-steroidogenic cells, presumably macrophages, but not in luteal cells (F). Arrows indicate positive staining. NC: negative control. Scale bar– 50 μm.

The immunohistochemistry assay localized active CASP3 in luteal cells. The pattern differed individually for each animal and overall the expression of CASP3 protein could be found in at least one sample of every stage analyzed (Fig 2A–2D), except for CA (Fig 2E). Non-steroidogenic cells, presumably macrophages, were the only cells stained for TNF antibody in the CL (Fig 2F). The staining was rare and consistent with the results on mRNA level.

### 3.2. Lynx

#### 3.2.1 Iberian lynx, frCL *vs*. perCL post-mating

**Fig 3:**
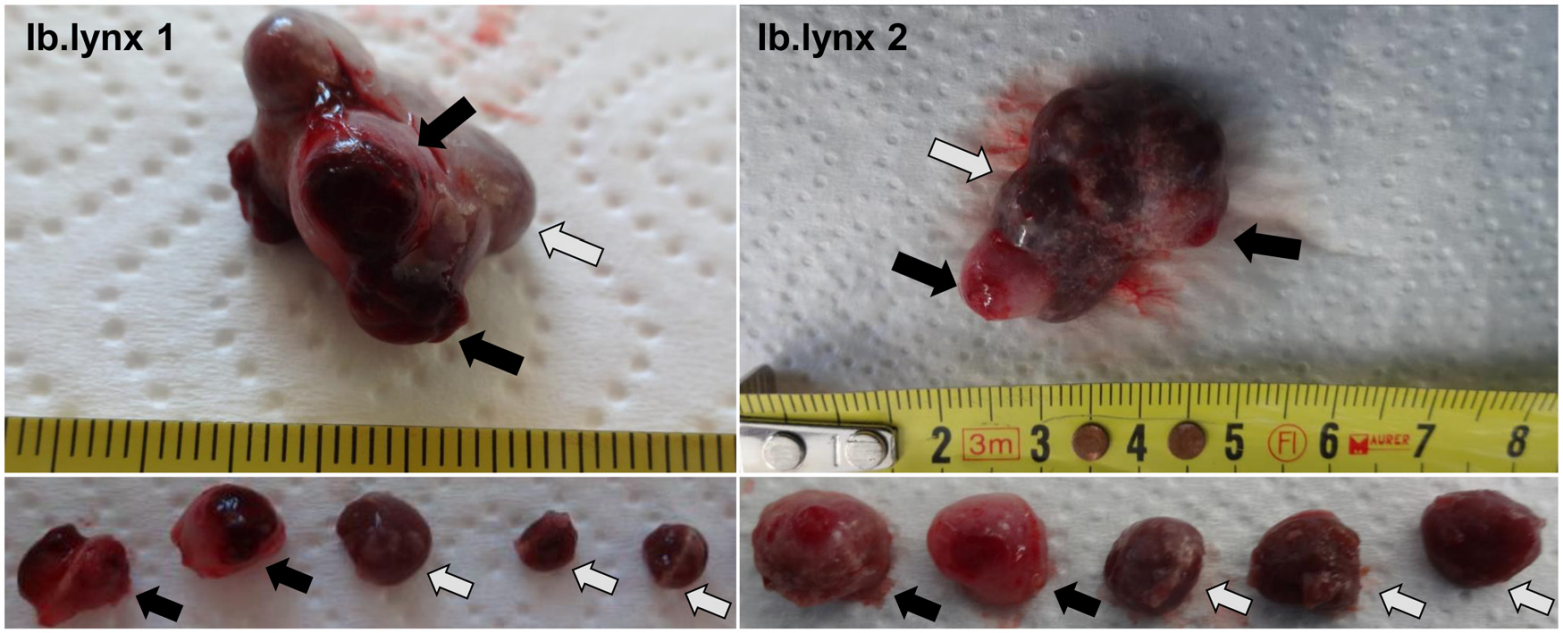
Morphological appearance of the Iberian lynx ovary. Ovaries of the Iberian lynx 1 and 2 (Ib.lynx 1 and Ib.lynx 2, respectively), containing frCL and perCL. Black arrows indicate frCL with ovulation scars, gray arrows indicate perCL from previous cycle/s. Scale–mm.

**Fig 4:**
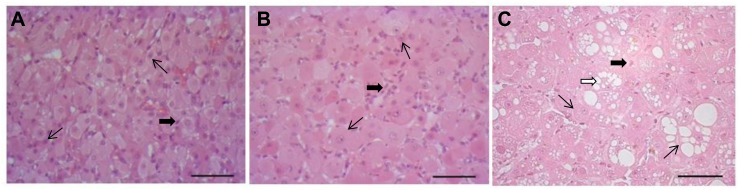
Haematoxylin and eosin stained sections of CL of Iberian and Eurasian lynx. A. Formation of the CL in Iberian lynx: frCL, seven days after natural mating. B. Maintenance of the CL in Iberian lynx: perCL of previous cycle/s after mating. C. Signs of regression in the CL of pre-mating Eurasian lynx: perCL of previous cycle/s during breeding season. Thin arrows indicate luteal cells. Thick black arrows indicate non-steroidogenic cells. White arrows indicate coarse vacuolation. Scale bar– 50 μm.

During dissection of ovaries, two types of CL could be clearly distinguished: frCL from a current ovulation and perCL from previous cycle/s (Fig 3). Fresh CL were paler and bigger than perCL and had an ovulation scar on their surface; perCL were smaller, darker and partly located inside of the ovary. Subsequent histologic analysis of these CL revealed a stage of formation for frCL and a stage of development/maintenance for perCL (Fig 4A and 4B). The formation stage of frCL exhibited processes of tissue reorganization, where predominantly small luteinizing cells varied in shape and size. The development/maintenance stage of perCL showed no signs of tissue regression; luteal cells were large and polyhedral, exhibiting fine vacuolation. The histomorphological analysis was done with reference to the established histologic staging on the domestic cat [24]. Hormone analysis revealed very high concentrations of estrogens in frCL of Iberian lynx and maintained concentrations of progesterone in perCL (Table 2).

**Table 2:** Intraluteal concentrations of estrogens and progesterone in Iberian and Eurasian lynx. Intraluteal concentrations of estrogens and progesterone in the domestic cat can be found in Amelkina *et al* [24]

Animal	Type of CL (n)	Estrogens, ng/gmean ± SD	Progesterone, μg/gmean ± SD
Iberian lynx 1	frCL (2)	6267.1, 5688.9	38.9, 31.8
	perCL (4)	241 ± 35	19.6 ± 5
Iberian lynx 2	frCL (3)	6517.6 ± 3795.3	20 ± 8.4 5
	perCL (4)	211.3 ± 201	16.3 ± 1.4
Eurasian lynx, n = 5	perCL (8)	10.1 ± 10.1	9.9 ± 5.8

**Fig 5:**
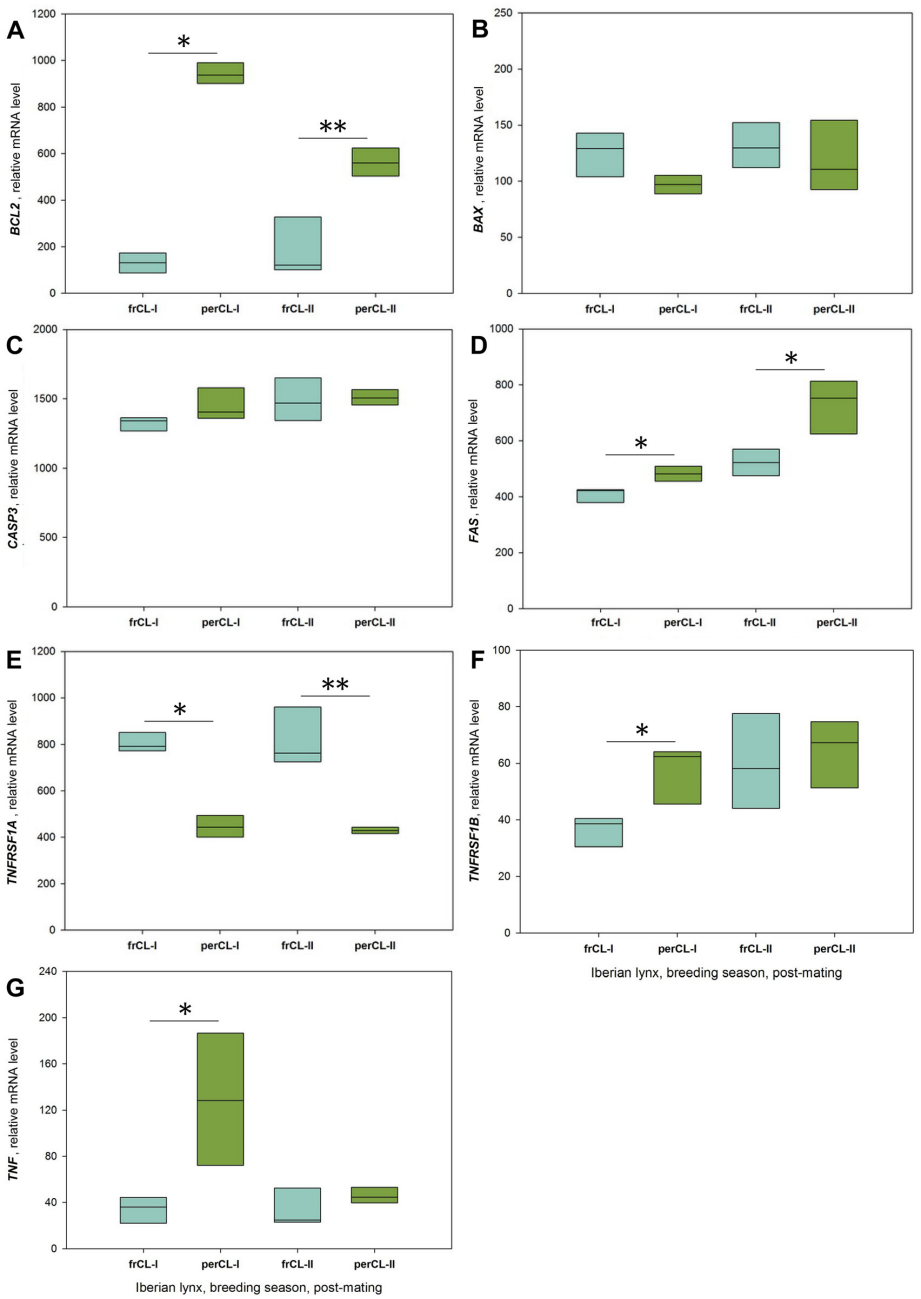
Intraluteal relative mRNA levels during the breeding season in post-mating Iberian lynx. Box plots depict the distribution of data; horizontal lines indicate the median. frCL-I/II: frCL from Iberian lynx1/2; perCL-I/II: perCL from Iberian lynx 1/2 * P < 0.05, ** P < 0.005; Mann-Whitney U-test.

All the factors examined were expressed in frCL and perCL of Iberian lynx. Relative mRNA levels of *BCL2* (P = 0.012, Iberian lynx 1; P = 0.0043, Iberian lynx 2; Fig 5A), *FAS* (P = 0.048, Iberian lynx 1; P = 0.0086, Iberian lynx 2; Fig 5D), *TNFRSF1B* (P = 0.01, Iberian lynx 1; Fig 5D) and *TNF* (P = 0.02, Iberian lynx 1; Fig 5G) were all higher in perCL compared to frCL (Fig 5). Relative mRNA levels of *TNFRSF1A* were higher in frCL compared to perCL (P = 0.01, Iberian lynx 1; P = 0.004, Iberian lynx 2; Fig 5E). In both animals, no significant changes in relative mRNA levels of *BAX* and *CASP3* were observed (Fig 5B/5C).

**Fig 6:**
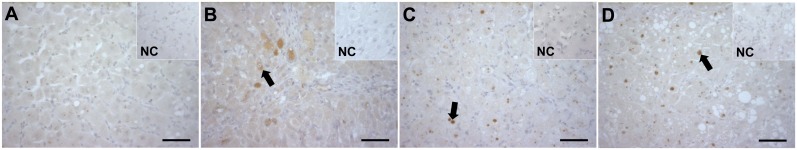
Immunohistochemical localization of CASP3 and TNF proteins in CL of Iberian and Eurasian lynx. A. Negative staining for active CASP3 in lynx CL tissue. B. frCL of Iberian lynx; strong staining for TNF protein in transforming cells (arrows). C. perCL of Iberian lynx; staining for TNF protein in non-steroidogenic cells, presumably macrophages (arrows). D. perCL of Eurasian lynx; staining for TNF protein in non-steroidogenic cells, presumably macrophages (arrows). Staining of droplets within the cells is unspecific. NC: negative control. Scale bar– 50 μm.

Active CASP3 protein could not be localized in the analyzed CL tissue (example of negative staining in Fig 6A). Staining for TNF protein was observed in non-steroidogenic cells, presumably macrophages, in both types of CL, but also in some transforming cells of frCL (Fig 6B and 6C).

#### 3.2.2 Eurasian lynx, perCL in breeding season before mating

Histomorphological analysis of perCL in Eurasian lynx revealed signs of luteal regression: coarse vacuolation could be observed across the luteal tissue (Fig 4D). This state was comparable to the histologic stage of early regression in domestic cat [24]. No CA stage of CL was observed. Concentrations of progesterone and estrogens were present in the luteal tissue (Table 2). All the factors examined were expressed in perCL of Eurasian lynx, including *BCL2* (relative mRNA level 726.4 ± 365.8), *BAX* (relative mRNA level 132.0 ± 46.9), *CASP3* (relative mRNA level 1092.3 ± 109.4), *FAS* (relative mRNA level 562.9 ± 193.7), *TNFRSF1A* (relative mRNA level 458.2 ± 115.2), *TNFRSF1B* (relative mRNA level 62.9 ± 33.1) and *TNF* (relative mRNA level 44.1 ± 19.0). Active CASP3 protein could not be localized in the analyzed CL tissue (example of negative staining in Fig 6A). Strong staining for TNF protein was identified in non-steroidogenic cells, presumably macrophages (Fig 6D).

## Discussion

Apoptosis has been shown to be highly involved in tissue remodeling during development and regression of the CL in a variety of species [8, 63], but no research has been done in the feline luteal phase. In our study, the mRNA of all factors analyzed was present in the CL tissue of domestic cat and lynx throughout all stages. We showed a significant change in the expression of *BCL2*, *CASP3*, *TNFRSFB1* and *FAS* throughout luteal stages in the domestic cat, what might indicate their potential involvement in the regulation of the feline luteal phase. The significantly elevated mRNA level of *TNFRSF1A* in both the domestic cat and the Iberian lynx during CL formation may suggest a possible conserved involvement of this factor in tissue reorganization and luteinization after ovulation. In contrast, the CL capacity to express *TNF* differed considerably between domestic cat and both Iberian and Eurasian lynx. The presence of mRNA of pro-apoptotic factors *BAX*, *CASP3*, *TNFRSFR1* and *FAS* in perCL of Iberian and Eurasian lynx suggests potential capacity of perCL to undergo apoptosis. Significantly higher expression of pro-survival factors *BCL2* and *TNFSFR1B* in perCL compared to frCL of Iberian lynx may be part of the mechanism to secure the structural integrity of perCL. Histologic analysis revealed a different structural state of perCL in Iberian and Eurasian lynx during the pre- and post-mating periods, respectively.

Gene expression of pro-apoptotic factors *BAX* and *CASP3* was found in CL throughout the whole luteal phase of the domestic cat. From the mRNA levels of *BAX* and *CASP3* analyzed in this study, it is not clear whether the protein product is activated; therefore, the data cannot indicate whether the apoptotic signal has been implemented. However, active CASP3 protein was detected in domestic cat luteal tissue by immunohistochemistry, showing local apoptotic processes in reorganization (early stages) and regression (late stages) of feline CL. Likewise, CL of other species express BAX and CASP3 at various stages [35, 36, 38, 47, 64–66]. Fas ligand could not be identified in the tissues studied and therefore was not included in the gene analysis. However, its receptor, *FAS*, was present in domestic cat CL and was constantly expressed throughout the non-pregnant luteal phase. In pregnancy, the regressing CL of the domestic cat showed higher expression of *FAS* compared to its previous stage, indicating a possible involvement of the FAS/Fas ligand system in regulating feline luteolysis during pregnancy. Likewise, the increase in FAS and Fas ligand expression with regression of the CL has been shown in many species, including rodents and cattle [7, 42, 67].

In this study, luteal cells of the domestic cat, while presenting high receptivity to TNF *via* expression of *TNFRSF1A* and *TNFRSF1B*, were not identified as a source of this cytokine, in contrast to other species [43, 53, 68]. The feline TNF may be derived from other sources, such as immune and endothelial cells [43]. In both rat and bovine CL, TNFRSF1A has been associated with the processes of luteolysis [48, 51]. In our study, the relative mRNA level of *TNFRSF1A* was significantly higher during the CL formation stage in both the pregnant and non-pregnant luteal phases of the domestic cat. Moreover, such increased expression was also noted in the forming frCL of the Iberian lynx. We hypothesize that this receptor is involved in processes of CL formation and may be conserved for both domestic cat and Iberian lynx. The involvement of TNFRSF1A in CL formation can be as an apoptotic mediator of the tissue reorganization processes.

For the domestic cat, the process of luteolysis and differences in it between pregnancy and the non-pregnant luteal phase are not yet known. The CL of infertile cycles could regress in response to active luteolytic signals, like in sheep and rat [8, 63], or undergo a passive regression, like in dogs [69]. Based on our results, we hypothesize that the life span of CL of pregnancy is supported by luteotropic factors. The CL of non-pregnant luteal phases, therefore, may passively regress due to the absence of such support. These CL may still produce receptors potent for survival like in pregnancy (*TNFRSF1B* expression), but either lack the activation of the survival cascade by ligands or have the luteotropic signal blocked on the downstream level. This may also explain high variations in reported functional life spans of CL in non-pregnant luteal phases in domestic cats, *e*.*g*., from 26 to 62 days as evidenced by serum progesterone levels [22, 23].

The histomorphology of Iberian lynx CL is described for the first time in this study. Due to the histomorphological similarity of Iberian lynx CL to the domestic cat, it was possible to distinguish between frCL undergoing formation and structurally maintained perCL by histologic analysis. Interestingly, perCL of Eurasian lynx before mating exhibited structural regression signs, while perCL of Iberian lynx after mating showed no signs of regression. Moreover, all of the perCL analyzed showed functional activity, as evidenced by intraluteal progesterone and estrogen concentrations. Histologic observations allow us to hypothesize that the state of perCL changes under the influence of new ovulations, returning it to the state of maintenance from regression. This is supported by reports on the bobcat, in which structurally persistent CL were responsive to gonadotropin treatment, *i*.*e*., exhibited elevation in progesterone secretion [14].

In our study, we could only collect samples from two Iberian lynx, due to the critically endangered status of these species. The observations are, therefore, limited to the system in two animals; however, they allow us to partly answer introduced questions and establish the direction of further studies. The detection of pro-apoptotic factors *BAX*, *CASP3*, *TNFRSF1A* and *FAS* at the mRNA level in all CL analyzed suggests that perCL, although structurally persistent, have the capacity to undergo apoptosis. The next question would therefore be whether CL persist due to the absence of luteolytic signal or because such luteolytic signal is further blocked, supposedly *via* involvement of luteotropic factors. Based on our results, we suggest the second mechanism. Here, the candidate for the rescue factor is BCL2, which in higher concentrations would outcompete BAX for BH3-domain binding and thus either prevent BAX from activating or, if already activated, from proceeding to MOM permeabilization [70]. Additionally, TNFRSF1B may compete for the ligand binding with TNFRSF1A, resulting in the activation of pro-survival rather than pro-apoptotic pathways. Both these pro-survival factors, *BCL2* and *TNFRSF1B*, were present at the mRNA level in perCL of Eurasian lynx and significantly higher expressed in perCL compared to frCL in analyzed Iberian lynx.

The phenomenon of physiological CL persistence is not common among mammalian species. So far, Eurasian, Iberian and Canada lynx are the only species where both structural and functional persistence of CL has been reported. In cows, the persistence of such functional CL is viewed as a pathology, because it prevents the initiation of a new ovarian cycle [71]. The unique physiological persistence of CL in lynx offers opportunity to learn more on the mechanisms of CL rescue. For instance, many human clinical studies are focused on a search for rescue factors to prevent premature regression of the CL. Physiologically, such rescue occurs during pregnancy, *e*.*g*., in humans by chorionic gonadotropin [72, 73], or *post-partum* in lactating rats [74, 75]. In baboons, the administration of human chorionic gonadotropin or gonadotropin releasing hormone prolonged the life of CL even during the early follicular stage [76]. In all cases, rescue of the CL is accompanied by increased progesterone concentrations. Telleria suggests that regression of the CL can be interrupted and even reversed, either by rescuing its capacity to produce progesterone or by interfering with apoptosis [77]. As shown by previous studies [27] and by intraluteal hormone concentrations in our study, perCL of both Iberian and Eurasian lynx retain the capacity to produce progesterone. This characteristic could be the basis for the structural integrity of perCL and may involve potential luteal factors to ensure functional persistence. The elevation of pro-survival factors, such as *BCL2* and *TNFRSF1B*, could be a mechanism to prevent transduction of the apoptotic signal and rescue perCL from its structural demise. Interestingly, it is hypothesized that BCL2 is involved in prolongation of CL lifespan during pregnancy in humans [78]. Moreover, progesterone has been shown to promote BCL2 expression and decrease BAX to BCL2 ratio in bovine luteal cells [79]. It might be possible that the same mechanisms that rescue CL of the domestic cat during pregnancy, as discussed above, are involved in the physiological persistence of lynx CL. If this assumption is true, research on the luteotropic and luteolytic systems in the domestic cat becomes essential for understanding the unique reproduction of monoestrous lynx.

Interestingly, gene expression of the pro-apoptotic receptor *FAS* was significantly higher in maintained perCL compared to frCL of Iberian lynx. This observation leads to two possible scenarios: (i) either the elevated FAS indicates the increased susceptibility of perCL to apoptosis and with this the existence of a survival system to block the FAS-induced apoptotic signal, including the possible involvement of a soluble form of FAS [80]; or (ii) elevated FAS points to the pro-survival action of FAS itself. The latter is not unrealistic, because with the growing number of recent studies it is becoming clear that FAS can also exhibit diverse non-apoptotic actions depending on the tissue and conditions [81]. Consistent with this, elevated expression of FAS was found in early stage bovine CL compared to later stages [82]. In the second scenario, FAS might be one of the factors involved in the persistence of lynx CL and hence also one of the lynx reproductive characteristics divergent from those of the domestic cat. Another distinct characteristic of the lynx luteal system that was observed involves *TNF* gene expression. While mRNA levels of *TNF* were close to zero in the domestic cat CL, indicating an extra-ovarian source of this cytokine, in both Iberian and Eurasian lynx this factor was regularly expressed. Because the increased expression of *TNF* in perCL tissue coincided with a similar increase in *TNFRSF1B*, we suggest a local autocrine regulation of the perCL *via* this non-apoptotic receptor.

In summary, as in other species studied, apoptosis-related factors are expressed during different stages of luteal development and regression in the domestic cat CL. It appears that TNFRSF1A might play an important role during the formation of feline CL. In Iberian and Eurasian lynx, structurally intact perCL exhibited the potential capacity to undergo apoptosis. Of the factors studied here, we hypothesize that BCL2 and TNFRSF1B might play a luteotropic role and be involved in the protection of perCL from complete structural regression. Mechanisms by which apoptosis-related factors are involved in the feline luteal phase are uncertain. Our current work presents a basis for further research on apoptosis system in the feline luteal phase and indicates potential factors involved in the regression and persistence of feline CL.
